# Patterns of B‐cell lymphocyte expression changes in pre‐ and post‐malignant prostate tissue are associated with prostate cancer progression

**DOI:** 10.1002/cam4.7118

**Published:** 2024-03-25

**Authors:** Sudha M. Sadasivan, Ian M. Loveless, Yalei Chen, Nilesh S. Gupta, Ryan Sanii, Kevin R. Bobbitt, Dhananjay A. Chitale, Sean R. Williamson, Andrew G. Rundle, Benjamin A. Rybicki

**Affiliations:** ^1^ Department of Public Health Sciences Henry Ford Hospital Henry Ford Health + Michigan State University Health Sciences Detroit Michigan USA; ^2^ Department of Pathology Henry Ford Hospital Detroit Michigan USA; ^3^ Department of Pathology Cleveland Clinic Cleveland Ohio USA; ^4^ Department of Epidemiology, Mailman School of Public Health Columbia University New York New York USA

**Keywords:** benign biopsy, biochemical recurrence, CD20, CD3, CD4, inflammation

## Abstract

**Backround:**

Inflammation characterized by the presence of T and B cells is often observed in prostate cancer, but it is unclear how T‐ and B‐cell levels change during carcinogenesis and whether such changes influence disease progression.

**Methods:**

The study used a retrospective sample of 73 prostate cancer cases (45 whites and 28 African Americans) that underwent surgery as their primary treatment and had a benign prostate biopsy at least 1 year before diagnosis. CD3+, CD4+, and CD20+ lymphocytes were quantified by immunohistochemistry in paired pre‐ and post‐diagnostic benign prostate biopsy and tumor surgical specimens, respectively. Clusters of similar trends of expression across two different timepoints and three distinct prostate regions—benign biopsy glands (BBG), tumor‐adjacent benign glands (TAG), and malignant tumor glandular (MTG) regions—were identified using Time‐series Anytime Density Peaks Clustering (TADPole). A Cox proportional hazards model was used to estimate the hazard ratio (HR) of time to biochemical recurrence associated with region‐specific lymphocyte counts and regional trends.

**Results:**

The risk of biochemical recurrence was significantly reduced in men with an elevated CD20+ count in TAG (HR = 0.81, *p* = 0.01) after adjusting for covariates. Four distinct patterns of expression change across the BBG‐TAG‐MTG regions were identified for each marker. For CD20+, men with low expression in BBG and higher expression in TAG compared to MTG had an adjusted HR of 3.06 (*p* = 0.03) compared to the reference group that had nominal differences in CD20+ expression across all three regions. The two CD3+ expression patterns that featured lower CD3+ expression in the BBG compared to the TAG and MTG regions had elevated HRs ranging from 3.03 to 4.82 but did not reach statistical significance.

**Conclusions:**

Longitudinal and spatial expression patterns of both CD3+ and CD20+ suggest that increased expression in benign glands during prostate carcinogenesis is associated with an aggressive disease course.

## Graphical abstract

Longitudinal and spatial expression patterns of both CD3+ and CD20+ in pre‐malignant and malignant prostate were investigated in a cohort of patients with matched samples. The results suggest that increased expression in benign glands during carcinogenesis is associated with an aggressive disease course.

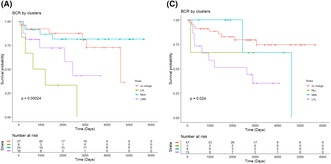

## 1 INTRODUCTION

In the prostate, inflammation has been observed in benign glands, glandular hyperplasia, and adenocarcinoma.^1^ There is growing evidence for a pro‐tumorigenic immune milieu that facilitates prostate cancer initiation and progression.^2^, ^3^, ^4^, ^5^ The immune response within the tumor microenvironment is critical for immune surveillance and evasion as well as response to treatment and drug resistance.^6^, ^7^ Although the infiltration of inflammatory cells might be associated with the proliferation of glandular cells, eventually leading to cancer development, the type of inflammatory environment, as defined by the amount and type of inflammatory cells, that most influence prostate cancer development and progression is unclear.

Benign regions of the human prostate commonly harbor inflammatory infiltrates that are highly heterogeneous in terms of location and extent as well as the type of immune cells.^8^, ^9^, ^10^ Inflammation in benign prostate has been shown to be predictive of higher PSA levels, higher Gleason score, and increasing odds of prostate cancer diagnosis.^11^, ^12^ However, other studies have found a reduced risk of prostate cancer associated with inflammation in the benign prostate.^13^, ^14^, ^15^ A better understanding of the immune cell environment in the benign prostate that precedes malignant transformation can provide insight into how the inflammatory response leading to carcinogenesis evolves and influences disease progression.

In contrast to the rich inflammatory environment characteristic of benign prostate, prostate cancers are generally not found to have the same level of inflammatory infiltrate. Most tumor‐infiltrating lymphocytes are T cells, with B cells accounting for a smaller proportion^16^, ^17^ and CD4+ T lymphocytes predominating over CD8+ T cells.^18^ In general, having more T cells in the tumor microenvironment portends to a better prognosis.^19^ In prostate cancer, the presence and extent of tumor‐infiltrating lymphocytes tend to correlate with clinical outcome^20^, ^21^, ^22^, ^23^ but the direction of the association is dependent on the composition of the lymphocyte subset.^9^ For example, CD4+ T cells have both shown a positive correlation, no correlation, and inverse correlation with Gleason score.^24^, ^25^, ^26^ The CD4+CD25+ subset of T cells known as regulatory T cells, when present at higher levels in tumor tissue and peripheral blood of men with prostate cancer appear to promote cancer progression.^27^ While CD20+ B‐cell tumor‐infiltrating lymphocytes have shown prognostic significance in several solid tumors,^28^, ^29^, ^30^ only a few studies have systematically quantified the presence of increased B cells in the prostate.^9^, ^31^, ^32^ CD20+ B‐cell expression appears to be greater in tumor compared to benign prostate with expression increasing or decreasing by Gleason grade depending on the type of B cell (naïve or memory).^9^ In terms of clinical outcome in prostate cancer, only a few studies have investigated CD20+ lymphocyte expression and have not found any association.^21^, ^33^

Most studies of human prostatic tissue comprise a snapshot in time, usually taken during prostate cancer surgery. While spatial comparisons can be made in such specimens regarding the immune environment in tumor and normal‐appearing adjacent tissue, such comparisons fail to capture the temporal evolution of the immune response in prostate cancer. We previously examined inflammatory markers in a cohort of men with paired prostate benign biopsies and subsequent prostate tumor and adjacent benign tissue and found that observation windows of both the pre‐ and post‐malignant prostate suggested that changing levels of M2 macrophages and GDF15 influences subsequent cancer progression.^34^ Using this same patient cohort, we now address the question of how changing levels of infiltrating B and T lymphocytes may influence prostate cancer progression by analyzing levels of CD3+, CD4+, and CD20+ positive cells in the benign prostate before a cancer diagnosis and in post‐diagnosis tumor and tumor‐adjacent benign regions of prostatectomy specimens.

## 2 MATERIALS AND METHODS

### 2.1 Study sample

After obtaining appropriate approval from the Henry Ford Health System Institutional Review Board, we ascertained 73 prostate cancer cases with a primary treatment of radical prostatectomy occurring between 1999 and 2012 who also had at least one previous benign prostate biopsy one or more years before their cancer surgery (Table 1). Prostate tumor and benign biopsy specimens were on average 1721.5 days (or 4.7 years) apart with a range of 368 days to 16.3 years. Cases were 38% African American (AA) and 62% White with an average age of 67.1 and 64.0 years at the time of diagnosis, respectively. About 30% of tumors were considered high grade (Gleason Grade group 3 and above) and 26% of patients in the cohort had a pathological stage of 3A and above (Table 1). To determine the date of biochemical recurrence (BCR) in cases, we electronically retrieved all PSA test results from the date of surgery forward. A total of 1423 PSA test results were retrieved, with the men in this sample having a median of 10 PSA tests post‐surgery and the number of tests ranging from two to 29. A BCR event was defined as having two consecutive detectable rising PSA levels (>0.2 ng/mL) 4 weeks or more after surgery. A total of 29% of cases experienced BCR with the median follow‐up of men who did not biochemically recur 7.4 years.

**TABLE 1:** Demographic and clinical characteristics of study sample by prostate cancer biochemical recurrence (BCR) status (*n* = 73).

Characteristic	No biochemical recurrence (*n* = 52)	Biochemical Recurrence (*n* = 21)	*p*‐Value
Race			0.98
White	32 (61.5%)	13 (62%)	
African American	20 (38.5%)	8 (38%)	
Mean age at diagnosis (years)	64 ± 7.07	67.05 ± 5.63	0.16
PSA at time of biopsy (ng/μL)	3.96 ± 15.28	7.31 ± 5.62	0.18
Mean number of PSA tests	6.63 ± 0.66	6.67 ± 0.81	0.98
Mean duration: Biopsy to surgery Dt (days)	1732.46 ± 175.89	1693.48 ± 244.89	0.90
Gleason grade group			
1	19 (36.5%)	1 (4.8%)	<0.001
2	24 (46.2%)	7 (33.3%)	
3	6 (11.5%)	10 (47.6%)	
4	2 (3.8%)	0 (0.0%)	
5	1 (1.9%)	3 (14.3%)	
Pathological stage			
2A	9 (17.3%)	0 (0.0%)	0.074
2B	15 (28.8%)	8 (38.1%)	
2C	18 (34.6%)	4 (19.0%)	
3A	8 (15.4%)	6 (28.6%)	
3B	2 (3.8%)	3 (14.3%)	

*Note*: *T*‐test was used for continuous and Chi‐square or Fisher exact test for categorical variables. For analysis, grade groups were categorized as 1 (Grade group 1–2) and 2 (Grade group 3–5), pathological stages were categorized as 1 (Pathological stage 2A–C) and 2 (Pathological stage 3A–C).

Abbreviations: BCR, biochemical recurrence; PSA, prostate‐specific antigen.

### 2.2 Specimen processing, pathology review, immunohistochemistry and image analysis

Surgical prostatectomy and biopsy specimens preserved as formalin‐fixed, paraffin‐embedded blocks were procured from the Henry Ford Hospital biorepository. For biopsy specimens used for analysis, we randomly selected a subset of blocks for each case from blocks with available tissue cores. For surgical prostatectomy specimen, one block per case with the primary tumor was selected. Serial sections at five‐micron thickness were cut from both the biopsy and the surgical prostatectomy specimens. The middle section was Hematoxylin & Eosin stained and used to confirm the tumor/benign status of the specimen by two independent pathologists. The remaining sections were stained for CD3, CD4, and CD20 cell markers using a standard immunohistochemistry (IHC) protocol. Slides were dried for 60 min in a 60°C drying oven, de‐paraffinized, and hydrated. Antigen retrieval was done using Agilent's FLEX High pH TRS (DM828) bath for 20 min at 97°C. Prediluted antibodies were used to differentiate immune cell types for Pan T‐cell lymphocyte marker (anti‐CD3+, Rabbit Polyclonal, Dako IR503), T helper lymphocyte (anti‐CD4+, Mouse Monoclonal 4B12, Dako IR649), and B‐cell lymphocyte marker (anti‐CD20+, Mouse Monoclonal L26, Dako IR604). The processed slides were loaded on Agilent's Dako Autostainer Link 48 (Dako North America, Carpinteria, CA) and incubated with FLEX Peroxidase Block (SM801) for 5 min. CD3+ and CD20+ staining involved incubation with primary antibody mix (anti‐CD3+ Rabbit polyclonal Dako IR503 or anti‐CD20+ Mouse monoclonal L26 Dako IR604) for 20 mins, FLEX/HRP labeled polymer (SM802) for 20 min, wash buffer (K8007) for 5 min, and FLEX DAB+ Sub‐Chromogen (SM803) for 10 min. CD4+ staining involved incubation with primary antibody (anti‐CD4+, Mouse Monoclonal 4B12, Dako IR649) for 20 min, followed by FLEX+ Mouse Linker (SM804) for 15 min, FLEX/HRP labeled polymer (SM802) for 15 min, Wash Buffer (K8007) for 5 min, and FLEX DAB+ Sub‐Chromogen (SM803) for 10 min. All slides were finally counterstained with FLEX Hematoxylin (SM806). The expression of CD3+, CD4+, and CD20+ immune cells were analyzed as single IHC staining on adjacent sections of the surgical and biopsy specimens. Matched surgical and biopsy specimens were processed together for IHC and slide imaging to maintain similar experimental conditions within the prostate benign biopsy–tumor pairs.

Whole slide scanning of CD3+, CD4+, and CD20+ stained matched benign biopsy and prostatectomy surgical slides were performed using a Ventana iScan HT slide scanner (Roche Diagnostics) and/or Aperio CS2 scanner at 40× magnification—the type of scanner did not vary within individual cases. Three glandular regions of interest that also included the surrounding stromal regions were delineated by the study pathologist: (1) benign glands on the biopsy slides (BBG); on prostatectomy slides (2) prostate malignant tumor glands (MTG) and; (3) tumor‐adjacent benign glands (TAG). Three to five high magnification (40×) images were captured from each of the three prostate regions—BBG, MTG, and TAG from comparable areas of CD3+, CD4+, and CD20+ stained slides. The captured images were then analyzed using ImageJ analysis software (https://imagej.nih.gov/ij/index.html) to deconvolute and separate the colors creating a binary image of unstained and stained tissue area enabling the quantification of CD3+, CD4+, and CD20+ cells. The automated application separates positive brown staining from the background stain, where the positive staining was defined as pixels with a brown staining optical threshold below 175 on the eight‐bit gray image. We also applied a size filter of 200 pixels for the brown staining to identify immune cells based on size and to eliminate areas of non‐specific staining. For each prostate tissue region, the count of CD3+, CD4+, and CD20+ stained cells was measured by utilizing a unitary cell size measure. To calculate the unitary cell size, approximately 3–5 representative images were selected for each of the three immune cell markers. The average of brown stained areas after applying the optical and size filter was ascertained for all stained tissue areas. This average value is referred to as the “unitary cell size”. The cell count for each immune marker was obtained by dividing the identified object area over the unitary cell size. The log‐transformed product of the count values was used for further analysis for each prostate tissue region of a study subject.

### 2.3 Statistical analysis

Differences in clinical and demographic characteristics of patients between biochemically recurrent and non‐recurrent cases were compared using chi‐square, Fisher exact, and independent sample t‐tests where appropriate. CD3+, CD4+, and CD20+ lymphocyte marker counts per unit of tissue region were log‐transformed to normalize the data. A Cox proportional hazard model was used to determine whether the different marker expression levels were associated with time to BCR. Time to BCR was defined as the duration between the date of surgery and the second PSA test that defined the recurrence event. Observation time for men who did not recur was censored at the date of the last observed post‐operative PSA test. Both unadjusted (only a covariate for the marker) and adjusted models were tested—the latter adjusted for race, Gleason grade group, and PSA level at the time of biopsy.

Expression patterns of T‐ and B‐cell markers across the three prostate regions of interest were clustered using TADPole (Time‐series Anytime DP).^35^ The optimal number of clusters was selected using the silhouette method, which calculates the silhouette coefficient for a range of cluster numbers.^36^ A higher coefficient represents more similarity between a sample and the other samples in the cluster. The optimal number of clusters is then selected which maximizes the silhouette coefficient. For each marker, the pattern with the least intraclass variability was set as the reference. Using Fisher's exact test, associations between clustered patterns and BCR, Gleason grade group, and pathological stage were tested. The association of time to BCR with the marker clusters was tested using a Cox proportional hazards model and subsequently visualized with Kaplan–Meier curves.

## 3 RESULTS

### 3.1 Demographic and clinical characteristics of the study cohort

Among our study sample of 73 prostate cancer surgical cases, 61.5% were White and the remaining 38.5% were African American (Table 1). Twenty‐eight percent of cases biochemically recurred in both whites (13 BCR cases out of 45 total) and African Americans (8 BCR cases out of 28 total). Study subjects had a median follow‐up after surgery of 3 years for biochemically recurrent and 7 years for non‐biochemically recurrent cases. The mean age at diagnosis was not significantly different between men who biochemically recurred (67.05 ± 5.63 years) and those who did not (64.0 ± 7.07 years). PSA at the time of benign biopsy was higher in men who biochemically recurred (7.31 ± 5.62 ng/μL) compared to men who did not (3.96 ± 15.28 ng/μL), but the difference was not statistically different (*p* = 0.18). There was no significant difference between the BCR groups for a mean number of PSA tests that were conducted between cohort entry and surgery with a mean of 6.63 ± 0.66 tests for those who did not biochemically recur versus 6.67 ± 0.81 for those patients that had biochemical recurrence (*p* = 0.98). Men who had biochemically recurrent disease had more severe disease with a greater percentage of Gleason grade group of 3 and above disease (61.9% vs. 17.2%; *p* ≤ 0.001) and pathological stage of 3A and above (42.9% vs. 19.2%; *p* = 0.04).

### 3.2 Expression levels of CD3 ^+^, CD4 ^+^ and CD20 ^+^ lymphocytes across prostate region

CD3^+^, CD4^+^, and CD20^+^ lymphocytes were expressed in all three prostate regions examined in the study—benign biopsy (BBG), tumor‐adjacent normal glands (TAG), and malignant tumor glands (MTG) (Figure S1). Overall, the expression levels of CD20^+^ were the lowest, followed by CD4^+^ with CD3^+^ having the highest expression (Figure S2A). When stratified on BCR status (Figure S2B), the expression levels of CD3^+^ did not vary significantly whereas expression levels for CD4^+^ and CD20^+^ cells were lower in men who had biochemically recurrent disease. The differences in expression of CD3^+^ or CD4^+^ markers between different regions of the prostate (BBG, TAG, or MTG) were not significant between patients with biochemically recurrent vs. non‐recurrent disease (Table 2), which did not change after controlling for race, PSA at entry, age at diagnoses and grade group. However, in unadjusted models, higher levels of CD20^+^ B‐cell lymphocyte expression were significantly associated with lower risk for BCR in the BBG (HR = 0.87, *p* = 0.04) and MTG (HR = 0.88, *p* = 0.04) regions of the prostate (Table 2). After controlling for race, PSA at cohort entry, age at diagnosis, and grade group, increased levels of CD20^+^ B‐cell lymphocyte expression were only significantly associated with lowered risk of BCR in the TAG region (HR = 0.81, *p* = 0.01).

**TABLE 2:** CD3^+^, CD4^+^, and CD20^+^ expression in the prostate regions and association to biochemical recurrence.

Marker region	Univariate Cox PH model		Multivariate Cox PH model^a^	
	HR (95% CI)	*p*‐Value	HR (95% CI)	*p*‐Value
CD3				
BBG	0.92 (0.77, 1.09)	0.36	0.96 (0.83, 1.19)	0.96
TAG	1.06 (0.74, 1.51)	0.76	0.86 (0.58, 1.28)	0.45
MTG	0.97 (0.77, 1.23)	0.82	1.02 (0.80, 1.31)	0.86
CD4				
BBG	0.93 (0.81, 1.08)	0.35	0.99 (0.86, 1.15)	0.90
TAG	0.98 (0.82, 1.17)	0.85	0.89 (0.73, 1.08)	0.22
MTG	0.88 (0.75, 1.05)	0.15	0.88 (0.72, 1.07)	0.20
CD20				
BBG	0.87 (0.76, 0.99)	0.04	0.90 (0.78, 1.03)	0.12
TAG	0.90 (0.77, 1.04)	0.15	0.81 (0.68, 0.95)	0.01
MTG	0.88 (0.76, 0.99)	0.04	0.94 (0.82, 1.08)	0.37

Abbreviations: BBG, benign biopsy; CI, confidence interval; HR, hazard ratio; MTG, malignant tumor glands; TAG, tumor‐adjacent benign glands.

^a^ Adjusted for race, PSA at entry, age at diagnoses, and grade group.

### 3.3 Patterns of CD3 ^+^, CD4 ^+^ and CD20 ^+^ expression in prostate regions and risk of BCR

Differences in CD3^+^, CD4^+^, and CD20^+^ expression between combinations of the three prostate regions of interest, BBG, TAG, and MTG, were not associated with risk of BCR for patients with or without adjusting for covariates such as race, PSA at entry, age at diagnoses and grade group (Table S1). Given the interdependence of each prostate region, we examined patterns of CD3^+^, CD4^+^, and CD20^+^ expression across all three regions to determine whether distinct expression pattern clusters were associated with aggressive disease. The TADPole method was used to cluster patients based on similar patterns of expression differences for each immune marker across the two prostate tissue samples for each matched patient (benign biopsy and prostatectomy) comprising of three defined regions of prostate (BBG‐TAG‐MTG). Using the silhouette method, an optimal number of four clusters, which maximized the silhouette coefficient was selected, for each of the three immune markers (Figure S3). Labels for each of the four clusters were based on the median of marker mean expression values in each of the three prostate regions (BBG, TAG, and MTG). Using the data for the mean expression values of each marker across all three tissue regions, tertiles were determined. If the median of the mean expression values for a marker in a specific region fell into the lowest tertile, then it was labeled as low (L), if the median fell into the middle tertile it was labeled as medium (M), and if the median fell into the highest tertile it was labeled as high (H). For instance, the CD4^+^ marker cluster depicted in the upper right panel of Figure S3B is labeled as “HLM” (high‐low‐medium). The median expression values of CD4^+^ for the three prostate regions in this cluster are 5.89, 3.50, and 4.99 and the tertile cutpoints are 3.78 and 5.59. Therefore, the first median falls into the “high” grouping, the second median falls into the “low” grouping and the third median falls into the “medium” grouping. For all three immune markers, the cluster showing the least absolute deviation across the three regions was used as the reference cluster for subsequent analyses (Figure S3A–C, Cluster “no change”).

**FIGURE 1:**
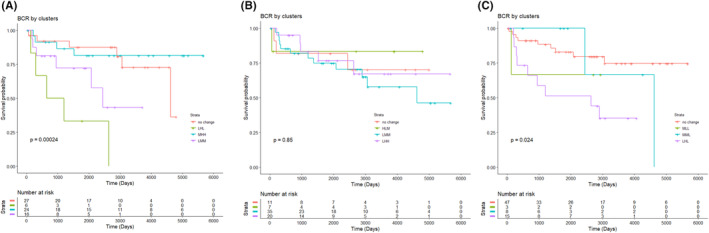
Risk to biochemical recurrence (BCR) for different clusters of patients based on CD3^+^ (A), CD4^+^ (B), and CD20^+^ (C) expression. A cluster is defined by a similar expression trend across the three regions of prostate‐benign biopsy (BBG), tumor‐adjacent benign glands (TAG), and malignant tumor glands (MTG) (as defined in Figure S3). Clusters with “no change” were the reference cluster for each of the markers. The tables below each plot indicate the numbers at risk in each of the clusters at each follow‐up period.

The demographic and clinical characteristics of patients by CD3^+^, CD4^+^, and CD30^+^ cluster classifications are detailed in Tables S2A–C, respectively. BCR and/or Gleason grade were the clinical characteristics that differed across the CD3^+^ and CD20^+^ clusters. For CD3^+^ expression clusters, the clusters with lower expression in BBG and higher expression in TAG and/or MTG had higher BCR rates and more advanced Gleason Grade when compared to reference cluster with similar expression levels across the three prostate regions (Table S2A). The BCR survival curves for four CD3^+^ clusters (Figure 1A) varied significantly (*p* = 0.00024) with men in the LHH cluster characterized as having low expression of CD3^+^ in BBG and higher expression in TAG and MTG having a marginally significant increased risk of BCR with an adjusted HR of 3.03 (*p* = 0.09) (Table 3). For CD4^+^ expression clusters, the clinical characteristics of the patients in the clusters were not statistically significant from the reference (Table S2B) and no difference in risk of BCR for patients in different clusters for CD4^+^ was observed when compared to the reference cluster (Table 3, Figure 1B). High expression of CD20^+^ in the TAG compared to the other two prostate regions discriminated the cluster with more advanced Gleason grade compared to the reference cluster with similar CD20^+^ expression levels across the three prostate regions (Table S2C). BCR survival curves for CD20^+^ clusters (Figure 1C) also varied significantly (*p* = 0.24) with the risk of developing biochemically recurrent disease 3 times higher for men in the LHL cluster (Table 3).

**TABLE 3:** Risk to biochemical recurrence for patients with different patterns of expression changes across the BBG‐TAG‐MTG region.

Marker cluster	Univariate Cox PH model		Multivariate Cox PH model^a^	
	HR (95% CI)	*p*‐Value	HR (95% CI)	*p*‐Value
CD3 (Cluster with no change is reference group, *n* = 27)				
Cluster LHL (*n* = 6)	7.52 (2.17, 26.15)	0.002	4.82 (0.73, 31.71)	0.10
Cluster MHH (*n* = 24)	0.66 (0.18, 2.40)	0.53	0.76 (0.20, 2.89)	0.69
Cluster LMM (*n* = 16)	2.82 (0.87, 9.11)	0.08	3.03 (0.86, 10.72)	0.09
CD4 (Cluster with no change is reference group, *n* = 11)				
Cluster HLM (*n* = 7)	0.62 (0.06, 5.95)	0.68	0.76 (0.07, 8.05)	0.82
Cluster LMM (*n* = 35)	1.30 (0.37, 4.63)	0.68	1.10 (0.29, 4.20)	0.89
Cluster LHH (*n* = 20)	0.97 (0.23, 4.07)	0.97	0.78 (0.17, 3.61)	0.76
CD20 (Cluster with no change is reference group, *n* = 47)				
Cluster MLL (*n* = 3)	2.19 (0.27, 17.51)	0.46	4.70 (0.54, 40.92)	0.16
Cluster MML (*n* = 8)	1.43 (0.31, 6.61)	0.65	1.45 (0.30, 7.04)	0.65
Cluster LHL (*n* = 15)	3.93 (1.52, 10.11)	0.005	3.06 (1.13, 8.26)	0.03

*Note*: CD3 Cluster groups: LHL—low, high, low; MHH—medium, high, high; LMM—low, medium, medium. CD4 Cluster groups: HLM—high, low, medium; LMM—low, medium, medium; LHH—low, high, high. CD20 Cluster groups: MLL—medium, low, low; MML—medium, medium, low; LHL—low, high, low.

Abbreviations: CI, confidence interval; HR, hazard ratio.

^a^ Adjusted for race, PSA at entry, age at diagnoses, and grade group.

## 4 DISCUSSION

The significance of immune cells in the tumor microenvironment in the initiation and progression of cancer has been well established.^37^ Our study aimed to understand whether the presence of B‐ and T‐cell lymphocytes in the pre‐ and post‐malignant prostate was associated with cancer progression. To meet this objective, we evaluated the levels of CD3^+^, CD4^+^ T‐cell lymphocytes, and CD20^+^ B‐cell lymphocytes in prostate cancer patients pre‐ and post‐malignancy. The pre‐malignant evaluation was based on a benign prostate biopsy (BBG) a year or more before the diagnosis of prostate cancer—the post‐malignancy evaluation was done with a matched prostatectomy specimen with pathologist‐defined tumor (MTG) and tumor adjacent normal (TAG) glandular regions. In this patient sample with sufficient follow‐up post‐prostatectomy to determine recurrence‐free survival, patients with biochemically recurrent disease had levels of CD20^+^ B cells and CD4^+^ T helper cells that were lower in all prostate regions when compared to CD3^+^ T cells. In addition, biochemically recurrent patients had levels of CD20^+^ and CD3^+^ cells that were higher in normal tumor‐adjacent glands (TAG) when compared to the malignant tumor glands (MTG) or the benign biopsy glands (BBG).

Other studies evaluating the immune infiltration in prostate tumor and adjacent normal tissue have had similar results.^9^ Wu et al. showed an overall higher level of total T cells compared to total B cells in both normal and prostate tumor tissue. While the authors observed increased levels of B cells in the tumor compared to the adjacent normal prostate, B‐cell numbers decreased with increasing Gleason grade. In our cohort, patients without biochemically recurrent disease had a similar pattern of increased levels of B cells in the tumor compared to benign environment. Among the various immune cell components that are critical in driving tumorigenesis, B‐cell lymphocytes have been shown to exhibit both tumor‐promoting and tumor‐inhibiting functions.^38^, ^39^ B cells are abundant in the tumor microenvironment and make up a significant portion of the tumor‐infiltrating lymphocyte population in many cancers. B cells in breast, colorectal, lung, and head and neck cancers are known to play a significant role in immune modulation and cancer progression.^38^, ^39^, ^40^, ^41^, ^42^, ^43^, ^44^, ^45^ The presence of B cells is generally associated with a good prognosis,^46^, ^47^, ^48^, ^49^ however, they also are associated with poor clinical outcomes for many cancer types.^50^ In breast cancer, regulatory B cells are associated with a poor prognosis when regulatory T cells are also present.^51^ The few studies that have evaluated B cells in prostate cancer progression suggest that the presence of B cells in prostate tumors does not alter disease outcomes.^33^, ^52^

The increased levels of B cells in the normal tumor‐adjacent glands of patients with aggressive disease suggest B cells may promote tumorigenesis. One of the key functions of B cells is to produce antibodies that can promote tumor development via secretion of tumor growth factors and suppress Th1 and CD8^+^ cytotoxic T cells.^38^, ^39^ B cells also produce lymphotoxin, a pro‐angiogenic factor that promotes tumor growth.^53^ Additionally, regulatory B cells (Bregs) also secrete cytokines such as TGFβ and interleukin‐10 (IL‐10) that can induce CD4^+^ T cells to suppress cytotoxic CD8^+^ T cells and natural killer (NK) cell function.^38^ Additionally, B cells are complex in nature with subtypes that are known to promote as well as inhibit carcinogenesis^38^ which could explain how B cells can either promote and/or restrict disease progression and subsequent disease prognosis, depending on the particular B‐cell subtype that is active in the tumor and its microenvironment.

Furthermore, our analysis indicated that patients who had a higher level of CD20^+^ B cells in the MTG and TAG prostate regions when compared to the pre‐disease benign biopsies tend to have worse outcomes when compared to patients who had similar levels of these immune cells in all three prostate regions. This observation supports the hypothesis that B cells are playing a pro‐tumorigenic role in more aggressive diseases potentially by activating pro‐tumorigenic factors, possibly via Bregs, in the tumor microenvironment and preventing anti‐tumor activity by immune cells. Similar patterns of B‐cell expression have been observed by others. Woo et al.^32^ showed higher B‐cell infiltration in the intra‐tumoral cancer regions of the prostate when compared to the extra‐tumoral region for 53 prostatectomy specimens in a recurrence group subset of 26 patients indicating the potential of B‐cell activation of tumorigenesis. However, the authors did not have data on the immune cell profile of pre‐malignant benign biopsies for these patients. Hence, our study provides the additional information on the risk of recurrence based on the trends in the immune cell changes from a benign to an overt malignant prostate environment.

Overall, the levels of CD3^+^ cells were higher in the prostate regions examined compared to CD4^+^ and CD20^+^. Increased levels of T lymphocytes in benign prostate have been previously documented.^16^, ^54^ Moser et al.^54^ showed a significantly larger number of T lymphocytes compared to B lymphocytes and macrophages in benign prostate biopsy specimens from patients that had elevated serum PSA levels. Hussein et al. showed significantly more CD3^+^ T cells and CD68^+^ macrophages compared to CD20^+^ B cells in all tissues analyzed including benign hyperplastic, normal, and tumor regions. Increased levels of immune cells in the hyperplastic prostatic tissue adjacent to the tumor have been previously reported by other studies.^16^, ^54^ Hussein et al.^16^ showed significantly more immune cells (CD3^+^, CD20^+^, and CD68^+^) in benign hyperplastic regions of the prostate compared to normal and tumor regions. However, it is not clear if the normal regions analyzed by the authors were adjacent to the tumor. Moreover, the authors did not stratify the patients by their biochemical recurrence status. Patients with increased CD3^+^ expression in TAG and MTG compared to BBG had a significantly increased risk of BCR compared to patients who had unchanging levels of T cells across prostate regions. Earlier studies evaluating the role of CD3^+^ T cells in prostate cancer recurrence have shown similar results with increased levels of T cells (mainly CD3^+^ CD8^−^FoxP3^−^ T_helper_) in the tumor compared to the benign stroma of prostatectomies associated with recurrence even after controlling for clinical variables.^3^ Flammiger et al.^33^ studied the role of tumor‐infiltrating lymphocytes in prostate cancer and found that very low and very high levels of CD3^+^ T cells in tumor were associated with a shortened time to recurrence. However, the level of the lymphocytes in tumor‐adjacent benign tissue was not defined. B lymphocyte cells are known to influence T‐cell differentiation via secretion of cytokines such as TGFβ and Interleukin‐10,^55^, ^56^ which further induces Tregs, and their coexistence has been associated with worse outcomes.^51^ Further studies are needed to evaluate the coexistence of T cells and B cells in the prostate to understand how their combined presence might influence prostate cancer risk.

Our study utilized a unique cohort of patients who had matched benign biopsy and prostatectomy samples allowing us to understand how changes in the immune cell population influenced the development of aggressive disease. We were able to show that patterns of lymphocyte expression changes across regions of prostate were associated with aggressive disease and may represent an immunologic response to cancer development. As our study was observational, the timing and duration between pre‐diagnostic biopsy and post‐diagnostic surgery were not systematic but determined by clinical needs. Study patients had adequate follow‐up to ascertain disease recurrence events and benign biopsies were at least 1 year prior to diagnosis to minimize the potential of sampling of benign tissue from the prostate that already had a growing tumor. The sample size utilized for this analysis is also modest, which limits the precision of our risk estimations, yet, a racially heterogenous sample of patients increased the generalizability of our findings. One limitation of the study was the inability to evaluate regional co‐expression of T‐cell and B‐cell lymphocytes. However, the tissue sections utilized for each marker staining were adjacent to each other and therefore, provided expression levels within a thickness of only a few cells. The utilization of computer‐generated algorithm to quantify marker levels provided an unbiased quantitative score for each lymphocyte measure.

In conclusion, men with higher levels of CD3^+^ T cells and CD20^+^ B cells in prostate tumor and adjacent benign tissue compared to an earlier benign biopsy have a three to four‐fold increased risk of biochemically recurrent disease. Further dissection by time and space of the evolving immune environment in the prostate may allow for a better understanding of how aggressive disease develops and more importantly how to prevent it.

## AUTHOR CONTRIBUTIONS

**Sudha M. Sadasivan:** Conceptualization (equal); data curation (lead); formal analysis (equal); investigation (equal); methodology (equal); project administration (lead); resources (equal); software (supporting); supervision (equal); validation (supporting); visualization (equal); writing – original draft (equal); writing – review and editing (equal). **Ian M. Loveless:** Conceptualization (supporting); formal analysis (equal); methodology (equal); software (equal); validation (supporting); visualization (supporting); writing – original draft (supporting); writing – review and editing (equal). **Yalei Chen:** Conceptualization (supporting); formal analysis (equal); investigation (equal); methodology (supporting); resources (supporting); software (equal); supervision (supporting); validation (supporting); visualization (supporting); writing – review and editing (supporting). **Nilesh S. Gupta:** Conceptualization (supporting); investigation (equal); methodology (equal); project administration (equal); resources (equal); supervision (equal); visualization (equal); writing – review and editing (supporting). **Ryan Sanii:** Data curation (supporting); project administration (supporting); writing – original draft (supporting); writing – review and editing (supporting). **Kevin R. Bobbitt:** Conceptualization (supporting); investigation (equal); methodology (equal); resources (supporting); supervision (supporting); writing – review and editing (equal). **Dhananjay A. Chitale:** Conceptualization (supporting); investigation (equal); methodology (equal); resources (equal); supervision (supporting); visualization (equal); writing – review and editing (supporting). **Sean R. Williamson:** Conceptualization (supporting); investigation (equal); resources (equal); supervision (equal); visualization (equal); writing – review and editing (supporting). **Andrew G. Rundle:** Conceptualization (supporting); investigation (supporting); writing – review and editing (equal). **Benjamin A. Rybicki:** Conceptualization (lead); data curation (equal); formal analysis (equal); funding acquisition (lead); investigation (equal); methodology (equal); project administration (supporting); resources (equal); software (equal); supervision (lead); validation (equal); visualization (equal); writing – original draft (equal); writing – review and editing (equal).

## FUNDING INFORMATION

This study was supported by National Institutes of Health 5R01‐ES011126 and Henry Ford Hospital internal research funds.

## CONFLICT OF INTEREST STATEMENT

The authors do not have any competing interests.

## ETHICS STATEMENT

The tissue samples used in the study were part of the surgical consent obtained from the patients.

## Supporting information


**Figure S1.**.

**Figure S2.**.

**Figure S3.**.

**Table S1.**.

## Data Availability

The data that support the findings are available upon request from the corresponding author.
