# Supplementary figures and images for: Patterns of B‐cell lymphocyte expression changes in pre‐ and post‐malignant prostate tissue are associated with prostate cancer progression
Source: Cancer Med. 2024 Mar 25;13(6):e7118. doi: 10.1002/cam4.7118 (PMC10961600; doi:10.1002/cam4.7118)

## Slide 1
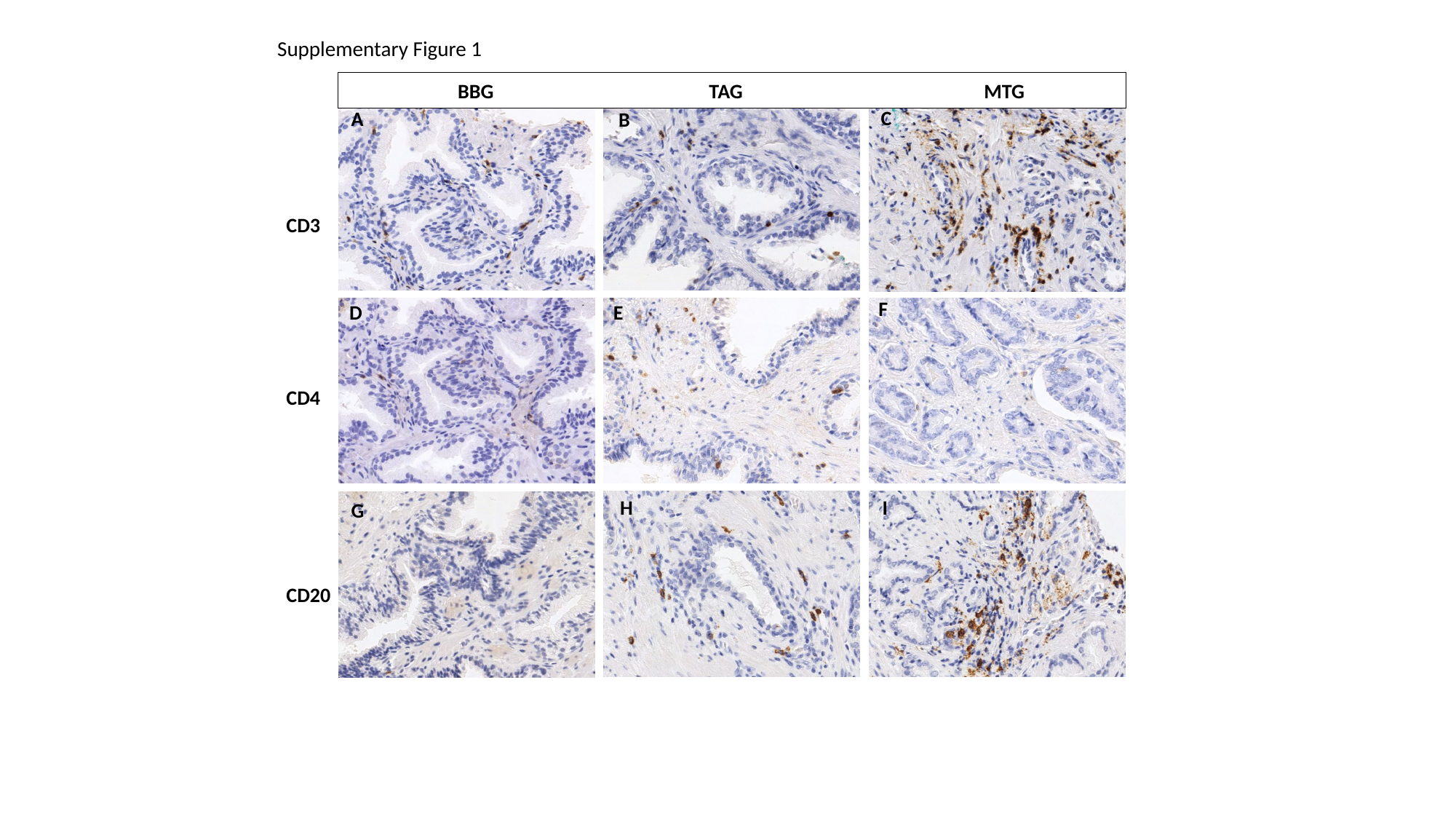

Supplementary Figure 1
	BBG		 TAG		 MTG
C
A
B
CD3
CD4
CD20
F
D
E
H
I
G

Supplement: Supplementary file 1 — Figure S1.. [file CAM4-13-e7118-s002.pptx]

## Slide 1
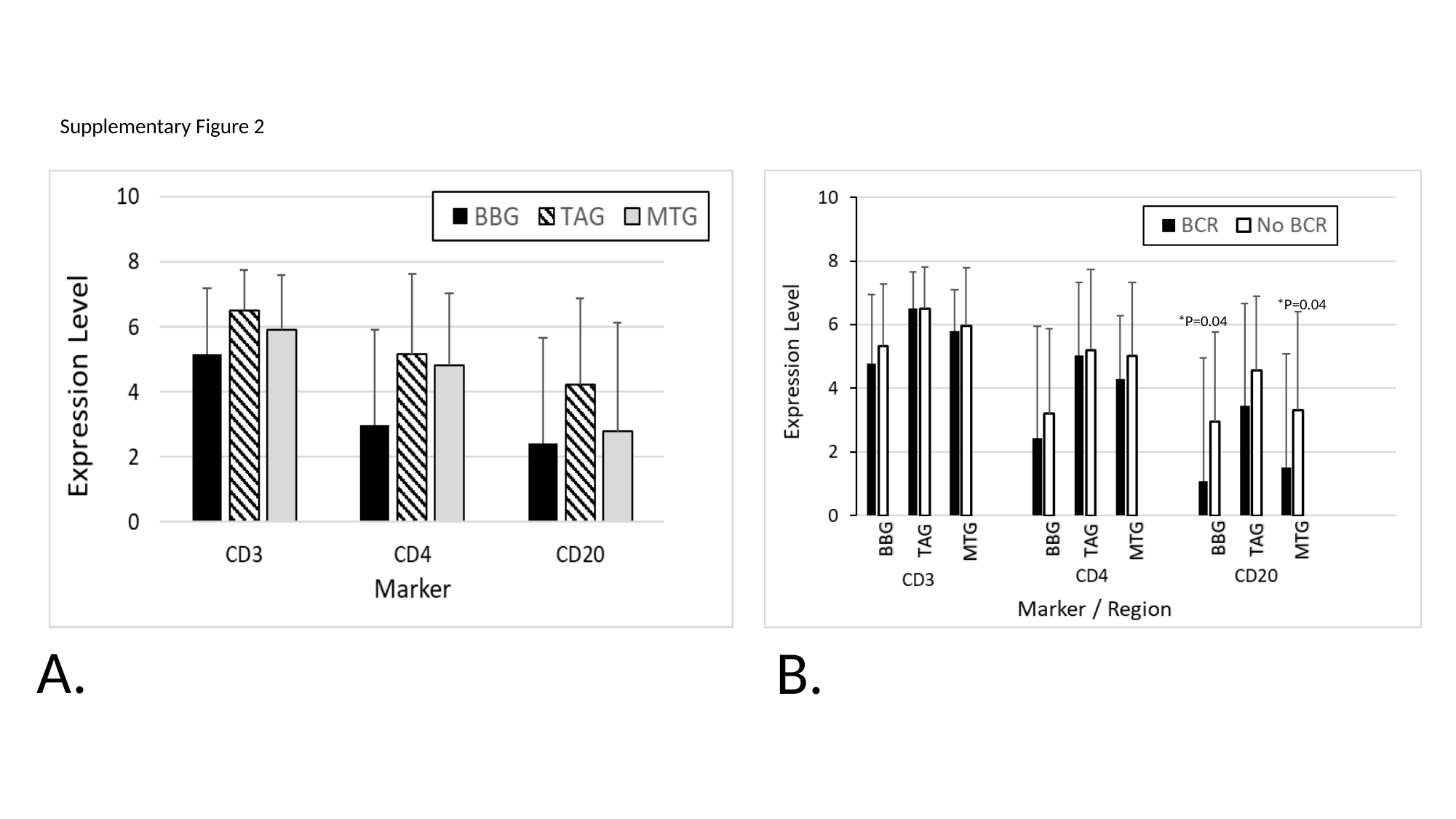

Supplementary Figure 2
*P=0.04
*P=0.04
A.
B.

Supplement: Supplementary file 2 — Figure S2.. [file CAM4-13-e7118-s004.pptx]
