# Supplementary material for: Patterns of B‐cell lymphocyte expression changes in pre‐ and post‐malignant prostate tissue are associated with prostate cancer progression
Source: Cancer Med. 2024 Mar 25;13(6):e7118. doi: 10.1002/cam4.7118 (PMC10961600; doi:10.1002/cam4.7118)
Supplement: Supplementary file 3 — Figure S3.. [file CAM4-13-e7118-s003.pptx]

## Slide 1
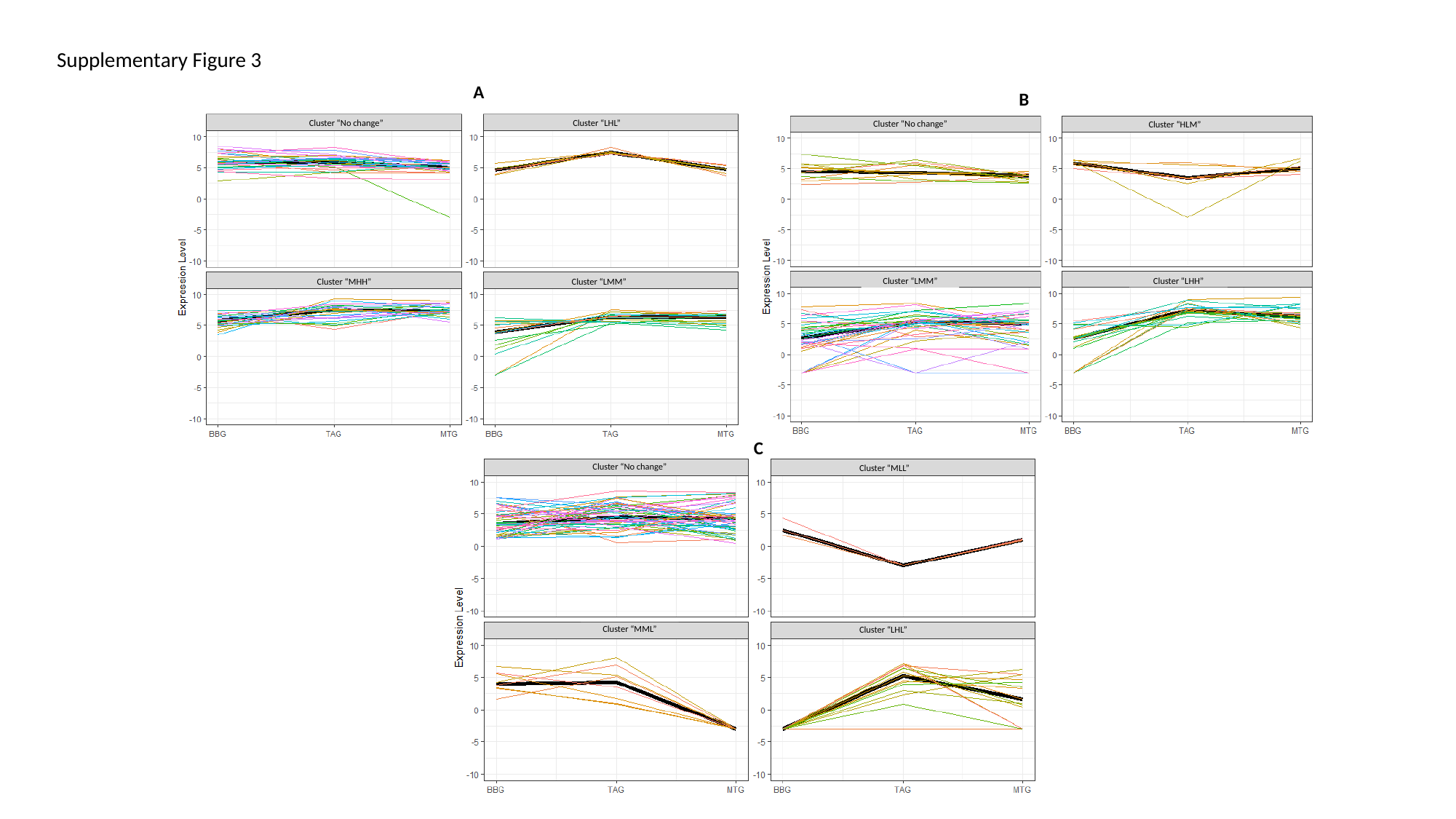

Supplementary Figure 3
A
B
Cluster “LHL”
Cluster “No change”
Cluster “No change”
Cluster “HLM”
Cluster “LHH”
Cluster “LMM”
Cluster “MHH”
Cluster “LMM”
C
Cluster “No change”
Cluster “MLL”
Cluster “MML”
Cluster “LHL”
