# Supplementary material for: Patterns of B‐cell lymphocyte expression changes in pre‐ and post‐malignant prostate tissue are associated with prostate cancer progression
Source: Cancer Med. 2024 Mar 25;13(6):e7118. doi: 10.1002/cam4.7118 (PMC10961600; doi:10.1002/cam4.7118)
Supplement: Supplementary file 4 — Table S1.. [file CAM4-13-e7118-s001.docx]

**Supplemental Table 1.** **CD3, CD4 and CD20 expression differences between prostate regions and association to biochemical recurrence.**

| **Marker Expression** | **Univariate Cox PH model** | | **Multivariate Cox PH model ^†^** | |
| --- | --- | --- | --- | --- |
|  | HR (95% CI) | P Value | HR (95% CI) | P value |
| **CD3** |  |  |  |  |
| **BBG-TAG** | 1.09 (0.94, 1.27) | 0.27 | 1.05 (0.88, 1.24) | 0.61 |
| **TAG-BBG** | 1.06 (0.91, 1.24) | 0.48 | 1.05 (0.91, 1.23) | 0.50 |
| **MTG-BBG** | 0.94 (0.74, 1.21) | 0.65 | 1.07 (0.79, 1.42) | 0.67 |
| **CD4** |  |  |  |  |
| **TAG-BBG** | 1.05 (0.93, 1.18) | 0.45 | 0.98 (0.86, 1.12) | 0.79 |
| **MTG-BBG** | 1.00 (0.88, 1.14) | 0.98 | 0.96 (0.83, 1.11) | 0.60 |
| **MTG-TAG** | 0.87 (0.70, 1.08) | 0.22 | 0.96 (0.76, 1.21) | 0.73 |
| **CD20** |  |  |  |  |
| **TAG-BBG** | 1.06 (0.96, 1.18) | 0.26 | 1.02 (0.90, 1.14) | 0.80 |
| **MTG-BBG** | 1.01 (0.91, 1.12) | 0.91 | 1.03 (0.92, 1.15) | 0.64 |
| **MTG-TAG** | 0.93 (0.83, 1.05) | 0.24 | 1.02 (0.88, 1.18) | 0.78 |
| **Abbreviations: BBG – Benign Biopsy, TAG – Tumor Adjacent benign glands, MTG – Malignant Tumor Glands, HR – Hazard Ratio, CI – Confidence Interval**  **^†^adjusted for race, PSA at entry, age at diagnoses and grade group** | | | | |

**Supplemental Table 2A. Demographic and clinical characteristics for patients in the different clusters of CD3 expression patterns**

| **Immune marker: Variable** | **Clusters** | | | |  | |  |
| --- | --- | --- | --- | --- | --- | --- | --- |
|  | **No Change** | **LHL** | **MHH** | **LMM** | |  | |
| **CD3** | n=27 | n=6 | n=24 | n=16 | | **P value** | |
| **Race** |  |  |  |  | |  | |
| White | 17 (63.0%) | 3 (50.0%) | 15 (62.5%) | 10 (62.5%) | | 0.95 | |
| African American | 10 (37.0%) | 3 (50.0%) | 9 (37.5%) | 6 (37.5%) | |  | |
| **Biochemical Recurrence** |  |  |  |  | |  | |
| No | 21 (77.8%) | 1 (16.7%) | 20 (83.3%) | 10 (62.5%) | | 0.01 | |
| Yes | 6 (22.2%) | 5 (83.3%) | 4 (16.7%) | 6 (37.5%) | |  | |
| **Gleason Grade Group** |  |  |  |  | |  | |
| 1 | 11 (40.7%) | 0 (0.0%) | 7 (29.2%) | 2 (12.5%) | | 0.01 | |
| 2 | 11(40.7%) | 0 (0.0%) | 12 (50.0%) | 8 (50.0%) | |  | |
| 3 | 4 (14.8%) | 3 (50.0%) | 4 (16.7%) | 5 (31.3%) | |  | |
| ≥4 | 1 (3.7%) | 3 (50.0%) | 1 (4.2%) | 1 (6.3%) | |  | |
| **Pathological stage** |  |  |  |  | |  | |
| 2A | 4 (14.8%) | 0 (0.00%) | 4 (16.7%) | 1 (6.3%) | | 0.14 | |
| 2B | 9 (33.3%) | 1(16.7%) | 10 (41.7%) | 3 (18.8%) | |  | |
| 2C | 9 (33.3%) | 1(16.7%) | 6 (25.0%) | 6 (37.5%) | |  | |
| 3A | 4 (14.8%) | 1(16.7%) | 3 (12.5%) | 6 (37.5%) | |  | |
| 3B | 1 (3.7%) | 3 (50.0%) | 1 (4.2%) | 0 (0.00%) | |  | |

**Abbreviations:**

**LHL – Low, High, Low; MHH – Medium, High, High; LMM – Low, Medium, Medium**

**Supplemental Table 2B. Demographic and clinical characteristics for patients in the different clusters of CD4 expression patterns**

| **Immune marker: Variable** | **Cluster** | | | |  |
| --- | --- | --- | --- | --- | --- |
|  | **No change** | **HLM** | **LMM** | **LHH** |  |
| **CD4** | n=11 | n=7 | n=35 | n=20 | **P value** |
| **Race** |  |  |  |  |  |
| White | 6 (13.3%) | 3 (6.7%) | 26 (57.8%) | 10 (22.2%) | 0.17 |
| African American | 5 (17.9%) | 4 (14.3%) | 9 (32.1%) | 10 (34.7%) |  |
| **Biochemical Recurrence** |  |  |  |  |  |
| No | 8 (15.4%) | 6 (11.5%) | 23 (44.2%) | 15 (28.9%) | 0.79 |
| Yes | 3 (14.3%) | 1 (4.8%) | 12 (57.1%) | 5 (23.8%) |  |
| **Gleason Grade Group** |  |  | | | |
| 1 | 4 (20.0%) | 2 (10.0%) | 8 (40.0%) | 6 (30.0%) | 0.98 |
| 2 | 3 (9.7%) | 4 (12.9%) | 16 (51.6%) | 8 (25.8%) |  |
| 3 | 3 (18.8%) | 1 (6.25%) | 8 (50.0%) | 4 (25.0%) |  |
| ≥4 | 1 (16.7%) | 0 (0.0%) | 3 (50.0%) | 2 (33.3%) |  |
| **Pathological stage** |  |  | | | |
| 2A | 1 (11.1%) | 0 (0.0%) | 6 (66.7%) | 2 (22.2%) | 0.94 |
| 2B | 4 (17.4%) | 2 (8.7%) | 12 (52.2%) | 5 (21.7%) |  |
| 2C | 2 (9.1%) | 2 (9.1%) | 10 (45.5%) | 8 (36.4%) |  |
| 3A | 3 (21.4%) | 2 (14.3%) | 5 (35.7%) | 4 (28.6%) |  |
| 3B | 1 (20.0%) | 1 (20.0%) | 2 (40.0%) | 1 (20.0%) |  |

**Abbreviations:**

**HLM – High, Low, Medium; LMM – Low, Medium, Medium; LHH – Low, High, High**

**Supplemental Table 2C. Demographic and clinical characteristics for patients in the different clusters of CD20 expression patterns**

| **Immune marker: Variable**  **CD20** | **Clusters** | | | |  | |  |
| --- | --- | --- | --- | --- | --- | --- | --- |
|  | **No Change** | **MLL** | **MML** | **LHL** | |  | |
|  | n=47 | n=3 | n=8 | n=15 | | **P value** | |
| **Race** |  |  |  |  | |  | |
| White | 32 (63.6%) | 1 (50.0%) | 5 (50.0%) | 7 (58.3%) | | 0.33 | |
| African American | 15 (36.4%) | 2 (50.0%) | 3 (50.0%) | 8 (41.7%) | |  | |
| **Biochemical Recurrence** |  |  |  |  | |  | |
| No | 38 (74.5%) | 2 (50.0%) | 6 (100.0%) | 6 (50.0%) | | 0.02 | |
| Yes | 9 (25.5%) | 1 (50.0%) | 2 (0.00%) | 9 (50.0%) | |  | |
| **Gleason Grade Group** |  |  |  |  | |  | |
| 1 | 11 (34.5%) | 2 (0.0%) | 3 (25.0%) | 4 (0.00%) | | 0.003 | |
| 2 | 23 (38.2%) | 0 (0.0%) | 2 (75.5%) | 6 (58.3%) | |  | |
| 3 | 11 (18.2%) | 1 (100.0%) | 1 (0.00%) | 3 (33.3%) | |  | |
| ≥4 | 52(9.1%) | 0 (0.0%) | 2 (0.00%) | 2 (8.3%) | |  | |
| **Pathological stage** |  |  |  |  | |  | |
| 2A | 6 (14,5%) | 0 (0.00%) | 0 (0.0%) | 3 (8.3%) | | 0.50 | |
| 2B | 17 (36.4%) | 1 (0.00%) | 3 (25.0%) | 2 (16.7%) | |  | |
| 2C | 14 (27.3%) | 0 (50.0%) | 2 (50.0%) | 6 (33.3%) | |  | |
| 3A | 8 (14.5%) | 1 (50.0%) | 2 (25.0%) | 3 (33.3%) | |  | |
| 3B | 2 (7.3%) | 1 (0.00%) | 1 (0.0%) | 1 (8.3%) | |  | |

**Abbreviations:**

**MLL – Medium, Low, Low; MML – Medium, Medium, Low; LHL – Low, High, Low**
